# Burden, predictors and short-term outcomes of peripartum cardiomyopathy in a black African cohort

**DOI:** 10.1371/journal.pone.0240837

**Published:** 2020-10-21

**Authors:** Juliet Nabbaale, Emmy Okello, Davis Kibirige, Isaac Ssekitoleko, Joseph Isanga, Patience Karungi, Elias Sebatta, Zhang Wan Zhu, Annettee Nakimuli, John Omagino, James Kayima

**Affiliations:** 1 Division of Adult Cardiology, Uganda Heart Institute, Kampala, Uganda; 2 Department of Medicine, Uganda Martyrs Hospital Lubaga, Kampala, Uganda; 3 Statistics Unit, Medical Research Council/Uganda Virus Research Institute and London School of Hygiene and Tropical Medicine Uganda Research Unit, Entebbe, Uganda; 4 Department of Obstetrics and Gynaecology, Case Hospital, Kampala, Uganda; 5 Graduate Entry Medical School, University of Limerick, Limerick, Ireland; 6 Department of Obstetrics and Gynaecology, School of Medicine-Makerere University College of Health Sciences, Kampala, Uganda; The Pennsylvania State University, UNITED STATES

## Abstract

**Background:**

Peripartum cardiomyopathy (PPCM) is an idiopathic cardiomyopathy presenting with acute heart failure during the peripartum period. It is common in patients of African ancestry. Currently, there is paucity of data on the burden, predictors and outcomes of PPCM in Uganda. This study aimed to investigate the prevalence, predictors and six-month outcomes of PPCM in an adult cohort attending a tertiary specialised cardiology centre in Kampala, Uganda.

**Methods:**

This study consecutively enrolled 236 women presenting with features of acute heart failure in the peripartum period. Clinical evaluation and echocardiography were performed on all the enrolled women. PCCM was defined according to recommendations of the Heart Failure Association of the European Society of Cardiology Working Group on PPCM. Poor outcome at six months of follow-up was defined as presence of any of the following: death of a mother or her baby, New York Heart Association (NYHA) functional class III-IV or failure to achieve complete recovery of left ventricular function (left ventricular ejection fraction ≤55%).

**Results:**

The median age, BMI and parity of the study participants was 31.5 (25.5–38.0) years, 28.3 (26.4–29.7) and 3 (2–4) respectively. The prevalence of PPCM was 17.4% (n = 41/236). Multiple pregnancy was the only predictor of PPCM in this study population (OR 4.3 95% CI 1.16–16.05, p = 0.029). Poor outcome at six-months was observed in about 54% of the patients with PPCM (n = 4, 9.8% in NYHA functional class III-IV and n = 22, 53.7% with LVEF <55%). No maternal or foetal mortality was documented.

**Conclusion:**

PPCM is relatively common in Uganda and is associated with multiple pregnancy. Poor outcomes especially absence of complete recovery of left ventricular function are also common. Large studies to further investigate long-term maternal and foetal outcomes in Uganda are justified.

## Introduction

Peripartum cardiomyopathy (PPCM) is an idiopathic cardiomyopathy presenting with acute heart failure following left ventricular (LV) systolic dysfunction towards the last months of pregnancy or few months post-delivery. It is a diagnosis of exclusion and the left ventricular ejection fraction (LVEF) is invariably <45% [1, 2].

PPCM has been documented to occur more commonly in women of African descent and is associated with poor outcomes [3–5]. Despite being a common cause of acute heart failure with reduced ejection fraction (EF) and associated devastating clinical outcomes in African women, there is limited published data about its burden, predictors and maternal or foetal outcomes in black African women outside Western and Southern Africa. The majority of published studies are from Nigeria [6–15] and South Africa [16–21].

Basing on hospital based studies, the incidence of PPCM in Africa is estimated to be 1 in 1000 live births in South Africa [20], about 1 in 96–2,700 deliveries in Nigeria [7, 8, 11] and 1 in 3,800 deliveries in Burkina Faso [22]. The prevalence of PPCM ranges between 4.1%-13.9% [12, 13, 23–25]. The reasons for this wide disparity in burden of PPCM in SSA has not been extensively studied but may be related to genetic and environmental differences.

The above studies have also demonstrated that PPCM is associated with poor maternal outcomes in African patients like high rates of maternal mortality, incomplete recovery of LV function and minimal improvement in New York Heart Association (NYHA) functional class after six to twelve months of follow-up [11, 17–19, 21, 22, 26].

Despite PPCM being frequently encountered in clinical care in Uganda, we lack contemporary information about its burden, predictors and related maternal or foetal outcomes. This study aimed to determine the prevalence of PPCM, its related predictors and six-month maternal and foetal outcomes in a cohort of women attending the adult cardiology clinic at Uganda Heart Institute, a tertiary specialised cardiology centre in Uganda.

## Materials and methods

### Study site and period

This study was conducted at the Uganda Heart Institute (UHI), Kampala Uganda. The UHI is an autonomous public tertiary institution that offers specialised paediatric and adult medical and surgical cardiology services. The UHI is part of the Mulago National Referral and Teaching Hospital Kampala, Uganda which is the main national referral hospital in Uganda offering tertiary specialty medical services with a bed capacity of 1,500.

The study was conducted from September 2018 to June 2019.

### Study design

This was a prospective study with a six-month follow-up period of all mothers with confirmed PPCM, to identify the pre-defined maternal and foetal outcomes of interest.

### Study eligibility

We included any woman referred from the different maternity health facilities who was ≥18 years presenting with symptoms of congestive cardiac failure that developed in the last month of pregnancy or during the first five months postpartum, without any identifiable cause for heart failure like coronary artery disease, pre-existing hypertension, rheumatic heart disease and congenital heart disease and offered written informed consent to participate in the study.

We excluded peripartum mothers with systolic blood pressure >160 or <95 mm Hg or diastolic >105 mm Hg, clinical conditions other than cardiomyopathy, significant liver disease (defined as liver transaminase levels >2 times the upper limit of normal), impaired renal function (defined as urea and/or creatinine >1.5 times the upper limit of normal) and pre-existing clinical conditions like ischemic heart disease, rheumatic heart disease, malignancy, congenital heart disease and known cardiomyopathies.

### Data collection

A pre-tested study questionnaire was used to collect all the significant socio-demographic, clinical and laboratory data of all the study participants. An electrocardiography (ECG) and echocardiography (ECHO) were performed on each study participant using standard criteria and methods.

PPCM was defined according to the Working Group on PPCM of the Heart Failure Association (HFA) of the European Society of Cardiology (ESC) recommendation of: an idiopathic cardiomyopathy presenting with heart failure secondary to LV systolic dysfunction (LVEF *<*45%) towards the end of pregnancy or in the months following delivery, if no other cause of heart failure is found [2]. Information about other echocardiographic variables like dimensions of the inter-ventricular septum (IVS), LV diameter in diastole (LVd), LV diameter in systole (LVs), left atrium (LA), right ventricle diameter in diastole (RVd), left ventricular post wall diameter in diastole (LVPWd) and mitral valve E:A ratio were also obtained. An ECG was performed to document presence or absence of left bundle branch block (LBBB).

A detailed clinical examination was also performed for assessment of pallor of mucous membranes, pedal oedema, jugular venous distension, hepatomegaly, pulmonary rales and heart sounds.

After the physical examination and completion of the ECG and ECHO, each study participant with confirmed PPCM received health education about the medical condition and standard heart failure regimen. The latter was an angiotensin converting enzyme inhibitor (ACEI) or angiotensin II receptor blocker (ARB), a loop diuretic, an aldosterone antagonist (spironolactone) and a beta blocker for those in the postpartum period while those in the last month of pregnancy received only a loop diuretic and a beta blocker. Digoxin was prescribed only for mothers with atrial fibrillation.

A six-month follow-up visit was scheduled mainly for a clinical review to re-evaluate the NYHA functional class, to perform a repeat ECHO to assess change in LVEF and to document the maternal and foetal outcomes. Poor outcomes were death of the mother and/or her baby, mothers in NHYA functional class III and IV and incomplete recovery of LV function (LVEF ≤55%).

## Statistical analysis

Proportions and medians with interquartile ranges (IQR) were used to summarise the socio-demographic, clinical, laboratory, ECG and ECHO characteristics, as appropriate. Chi-square, Fisher’s test, Student’s t and Mann–Whitney tests were used to compare categorical and continuous variables, as appropriate. Both univariate analyses and multivariate logistic regression were performed to identify independent predictors of PPCM. All values were expressed with odds ratio (OR) and 95% confidence intervals (CI). A p value of <0.05 and a 95% CI not containing 1 were considered of statistical significance.

### Ethics approval

This study was approved by the School of Medicine Research and Ethics Committee Makerere University and the Uganda National Council for Science and Technology (UNCST). All study participants were treated according to the declaration of Helsinki and offered a written informed consent to participate in the study.

## Results

### Prevalence of PPCM among women presenting with acute heart failure in the peri-partum period

The median age, body mass index (BMI) and parity of the study participants was 31.5 (25.5–38.0) years, 28.3 (26.4–29.7) and 3 (2–4) respectively with about 9% of the study participants having a multiple pregnancy. PPCM was confirmed in 41 study participants, giving a prevalence of 17.4%.

### Socio-demographic characteristics of the study participants

The socio-demographic characteristics of the study participants are shown in Table 1.

**Table 1:** Baseline socio-demographic and clinical characteristics of the study participants.

**Variable**	**Median (IQR)**	**Confirmed PCCM**	**PCCM Absent**	**P-value**
		**(n = 41, 17.4%)**	**(195, 82.6%) Median (IQR)**	
		**Median (IQR)**		
Age, years	31.5 (25.5–38)	35 (27–39)	31 (25–38)	0.084
Parity	3 (2–4)	3 (2–6)	3 (2–4)	1.000
Weeks into pregnancy or postpartum	9 (7–13)	8 (6–14)	10 (7–13)	0.024
Body mass index in kg/m^2^	28.3 (26.4–29.7)	27.7 (26–29.7)	28.6 (26.4–29.8)	0.188
**Variable**	**N (%)**	**Confirmed PCCM**	**PCCM Absent**	**P-value**
		**(EF <45%) N (%)**	**(EF ≥ 45%) N (%)**	
**Age, years**				
18–35	137 (62.0)	21 (53.9)	116 (63.7)	
>35	84 (38.0)	18 (46.1)	66 (36.3)	0.248
**Parity**				
1	45 (19.4)	9 (21.9)	36 (18.9)	
2–4	140 (60.3)	21 (51.2)	119 (62.3)	
≥5	47 (20.3)	11 (26.8)	36 (18.9)	0.384
**Occupation**				
Un-employed	85 (36.6)	12 (29.3)	73 (38.2)	
Self -employed	69 (29.7)	14 (34.1)	55 (28.8)	
Unskilled	9 (3.88)	1 (2.4)	8 (4.2)	
Professional	21 (9.05)	9 (22.0)	12 (6.3)	
Skilled	48 (20.7)	5 (12.2)	43 (22.5)	0.027
**Education level**				
No formal education	22 (9.5)	1 (2.4)	21 (11.0)	
Primary	80 (34.5)	14 (34.2)	66 (34.6)	
Secondary	69 (29.7)	9 (21.9)	60 (31.4)	
Tertiary	61 (26.3)	17 (41.5)	44 (23.0)	0.055
**Smoking**				
No	228 (98.3)	39 (95.1)	189 (98.9)	
Yes	4 (1.7)	2 (4.9)	2 (1.1)	0.145
**Alcohol consumption**				
No	204 (87.9)	35 (85.4)	169 (88.5)	
Yes	28 (12.1)	6 (14.6)	22 (11.5)	0.599
**Multiple pregnancy**				
Yes	21 (9.1)	9 (21.9)	12 (6.3)	
No	210 (90.9)	32 (78.1)	178 (93.7)	0.004
**Diabetes**				
No	229 (98.7)	40 (97.6)	189 (98.9)	
Yes	3 (1.3)	1 (2.4)	2 (1.1)	0.444
**Cardiomyopathy in past pregnancy**				
No	227 (97.8)	38 (92.7)	189 (98.9)	
Yes	5 (2.2)	3 (7.3)	2 (1.1)	0.040
**Family history of heart disease.**				
No	195 (84.1)	38 (92.7)	157 (82.2)	
Yes	37 (15.9)	3 (7.3)	34 (17.8)	0.106
**Pre-eclampsia**				
No	231(99.6)	41(100.0)	190(99.5)	
Yes	1(0.4)	0(0.0)	1(0.5)	1.000
**HIV Status**				
Negative	173 (74.6)	29 (70.7)	144 (75.4)	
Positive	59 (25.4)	12 (29.3)	47 (24.6)	0.534
**Body mass index, kg m**^**2**^				
<25	27 (12.1)	7 (18.4)	20 (10.8)	
25–29	149 (66.5)	25 (65.8)	124 (66.7)	
≥30	48 (21.4)	6 (15.8)	42 (22.6)	0.329
**Signs of heart failure**				
**Pedal oedema**				
No	125 (53.8)	16 (39.0)	109 (57.1)	
Yes	107 (46.1)	25 (61.0)	82 (42.9)	0.035
**Bibasilar crepitation**				
No	101 (43.5)	17 (41.5)	84 (44.0)	
Yes	131 (56.5)	24 (58.54)	107 (56.0)	0.768
**Hepatomegaly**				
No	224 (96.5)	34 (82.9)	190 (99.5)	
Yes	8 (3.5)	7 (17.1)	1 (0.5)	<0.001
**NYHA Class (1-1V)**				
I	75 (33.2)	10 (24.4)	65 (35.1)	
II	100 (44.3)	18 (43.9)	82 (44.3)	
III	49 (21.7)	11 (26.8)	38 (20.5)	
IV	2 (0.9)	2 (4.9)	0 (0.0)	0.013
**ECG findings**				
LBBB Absent	136 (59.7)	21 (56.8)	115 (60.2)	
LBBB Present	92 (40.4)	16 (43.2)	76 (39.8)	0.695

NYHA- New York Heart Association, ECG- Electrocardiography, LBBB- Left bundle branch block.

Compared to study participants without PPCM, patients with PPCM were more likely to have a multiple pregnancy (21.9% Vs 6.3%, p = 0.004), to have a history of cardiomyopathy in the past pregnancy (7.3% Vs 1.1%, p = 0.040) and were less unemployed (29.3% Vs 38.2%, p = 0.027). Among the study participants, only one had confirmed pre-eclampsia but without PPCM.

### Laboratory and ECHO characteristics of the study participants

The laboratory and ECHO characteristics of the study participants are shown in Table 2.

**Table 2:** Laboratory and echocardiographic characteristics of the study participants.

		Confirmed PCCM	PCCM Absent	
		(n = 41, 17.4%)	(n = 195, 82.6%)	
Variable	Median IQR	Median (IQR)	Median (IQR)	P-value
**Laboratory characteristics**				
Albumin	40 (34–45)	40 (34–45)	40 (34–45)	0.338
ALT	47 (43–49)	44 (36–47)	47 (43–49)	0.004
AST	39 (36–46)	37 (33–43)	39 (37–46)	0.255
Creatinine	107 (99–112)	99 (88–109)	108 (99–112)	0.001
Urea	6.2 (5.1–6.9)	5.3 (3.9–7)	6.3 (5.3–6.9)	0.009
Serum sodium	141 (139–143.5)	140 (138–143)	142 (139–144)	0.010
Serum potassium	4.9 (4.5–5.2)	4.8 (4.5–5)	5 (4.5–5.3)	0.115
Random blood glucose	5.5 (4.8–6)	5.4 (4.9–5.9)	5.6 (4.8–6.1)	0.668
**Echocardiographic findings**				
RVd	2.7 (2.4–3)	3 (2.5–3.5)	2.7 (2.4–2.9)	<0.001
LVd	4.5 (4.1–5)	6.1 (5.3–6.5)	4.4 (4.1–4.8)	<0.001
LVs	2.8 (2.5–3.2)	4.8 (3.2–5.4)	2.7 (2.5–3)	<0.001
EA	1.2 (1–1.4)	1.4 (1–1.6)	1.2 (1–1.4)	0.055
IVSd	0.9 (0.8–1.0)	0.9 (0.7–0.9)	0.9 (0.8–1)	1.000
LVPWd	0.9 (0.7–0.9)	0.8 (0.7–0.9)	0.9 (0.8–1)	<0.001
LA	3.4 (3.1–3.8)	3.4 (3.1–3.7)	3.4 (3.1–3.7)	<0.001

ALT- Alanine transaminase, AST- Aspartate transaminase, RVd- Right ventricle diameter in diastole, LVd- Left ventricle diameter in diastole, LVs- Left ventricle diameter in systole, IVSd- Interventricular septal diameter in diastole, LVPWd- Left ventricle posterior wall diameter in diastole, LA- Left atrial diameter.

Patients with confirmed PPCM had significantly lower median serum alanine transaminase (ALT), creatinine, urea and sodium levels compared to those without PPCM. Considering the ECHO findings, patients with PPCM had significantly higher median values of RVd, LVd, LVs and a lower median LVPWD.

### Factors associated with PPCM

On multivariate regression, multiple pregnancy (OR 4.3 95% CI 1.16–16.05, p = 0.029) was the only independent predictor of PPCM in this study population.

### Outcomes among patients with confirmed PPCM at six-months of follow-up

Poor outcomes defined as presence of either: maternal death, NYHA functional class III and IV or incomplete recovery of LV function (EF ≤55%) were observed in 22 (53.7%) patients. Individually, four (9.8%) patients were in NYHA functional class III and IV and 22 (53.7%) patients had incomplete recovery of LV function at six-month follow-up period. Full recovery of the left ventricular function occurred in 19 (46.3%) patients. No maternal or foetal mortality was documented at the six-month follow-up time point.

**Fig 1:**
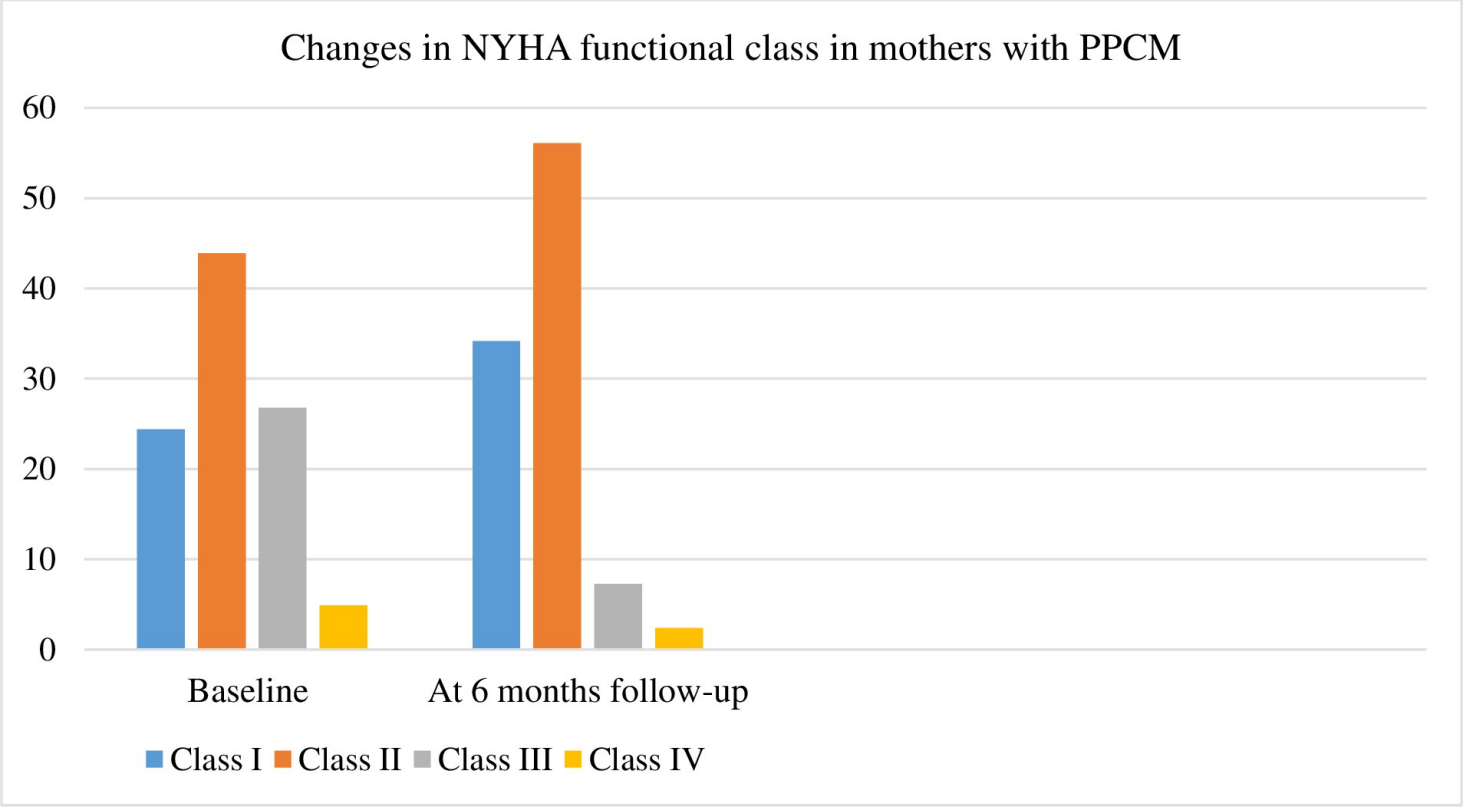
Changes in the New York Heart Association (NYHA) functional class in mothers with PPCM.

The changes in the NYHA functional class between baseline and at six months of follow-up are shown in Fig 1.

## Discussion

To our knowledge, this is the first study to robustly investigate the burden, predictors and short-term outcomes of PPCM in Uganda and Eastern Africa at large.

The reported study prevalence of PPCM of 17.4% among peripartum women is almost comparable to what has been reported in a study in South Africa (13.8%) [25] and Nigeria (13.9%) [13]. A lower prevalence of PPCM was reported in patients presenting with acute heart failure in a tertiary hospital in Botswana (4.1%) [23] and in adults patients referred for ECHO in rural Tanzania (7%) [24]. Another study conducted in Nigeria also reported a low prevalence of PPCM of 4.2%, which accounted for 52.4% of all ECHO confirmed diagnosis of cardiomyopathies [12].

Our study also demonstrated that the majority of the patients with PPCM had mild disease severity and a low prevalence of self-reported co-morbidities. About 31.7% (n = 13) of patients with PPCM presented in NYHA class III/IV in our cohort. This finding of mild disease severity in our study cohort starkly contrasts with what has been observed in other African studies. Studies conducted in Zimbabwe [26], Togo [27] and South Africa [17, 18, 21] reported about 46.5%, 77% and 66.7–89% of patients with PPCM presenting in NYHA functional class III/IV at baseline respectively. A high proportion of patients with PPCM presenting in NYHA functional class III or IV (68.8%) was also reported by a multi-country study investigating clinical characteristics of patients with PPCM in Africa, Middle East, Asia and Europe [28].

A self-reported diagnosis of diabetes mellitus and HIV infection was noted in 2.4% and 29.3% of the patients with PPCM respectively in our study cohort. Two studies conducted in South Africa reported varying prevalence of HIV of 10% [21] and 34% [18] in patients with PPCM.

No patient with PPCM in our study cohort was diagnosed with a thromboembolic event as part of the co-morbidities at baseline. PPCM is one of the recognised clinical causes of thrombo-embolic phenomenon in young women due to the related hypercoagulable state, venous stasis and diffuse vascular damage [29].

About 6.4% of patients with PPCM in the Peripartum Cardiomyopathy in Nigeria (PEACE) registry had an intra-cardiac thrombus on ECHO [8]. A comparable proportion of patients in thes

EURObservational Research Programme (EORP), an ongoing registry for patients with PPCM presented with thrombo-embolic events (6.8%) [28]. A higher prevalence of co-existing intra-cardiac thrombus of 9.3% and 16.7% was documented in a study in Zimbabwe [26] and South Africa respectively [21]. Thrombo-embolic events are also associated with a high risk of mortality. In a study conducted in Zimbabwe, deep vein thrombosis and an intra-mural thrombosis were presumed to be one of the direct causes of death in three patients with PPCM within 3 months of follow-up [26].

Multiple pregnancy was the only identified independent predictor of PPCM in our study population. It increased the odds of developing PPCM by 4-fold. PPCM occurs frequently in cases of multiple gestation status. According to one systematic review and meta-analysis, 9% of PPCM cases had multiple pregnancy [30].

Multiple pregnancy is associated with increased placental secretion of an anti-angiogenic factor called soluble fms-like tyrosine kinase-1 (sFlt-1) which causes wide-spread endothelial dysfunction and cardiac dysfunction [31].

There is limited data about predictors of PPCM in black African populations. The PEACE registry in Nigeria of 406 patients with PPCM identified lack of formal education, underweight, unemployment status, and pre-eclampsia as predictors of PPCM. The greatest association with PPCM was noted with underweight and pre-eclampsia which increased the odds of PPCM by 12- and 10-fold respectively. Lack of formal education and unemployed status increased the odds by 2- and 3 folds respectively [8].

At six months of follow-up, 53.7% of mothers in this study had incomplete recovery of LV function and 9.8% were in NYHA functional class III or IV. There was no documented maternal or foetal mortality in this study cohort.

There are several plausible explanations for these outcomes. A small proportion of mothers with PPCM presented in NYHA functional class III/IV (31.7%) at baseline which reflects a less severe disease. The follow-up period of the mothers with PPCM was also short. All patients with confirmed PPCM at presentation received health education about the condition and the significance of adherence to the prescribed standard heart failure regimen. Early initiation of optimal anti-failure therapy and good adherence directly translates to favourable clinical outcomes.

Varying rates of maternal mortality have been reported in most African studies. This could probably be due to differences in disease severity at diagnosis (low NYHA functional class and LVEF) and disparities in clinical management of PPCM during follow-up. Comparable rates of mortality of patients with PPCM at six months of follow-up were documented in Zimbabwe (11.6%) [26] and South Africa (10%, 12.6% and 13%) [17, 19, 25]. Higher rates of mortality were noted at six months of follow-up in Nigeria (24.2%) [11] and at 1 week post hospital discharge in Burkina Faso (48.3%) [22]. Predictors of mortality in the studies conducted in South Africa were low systolic blood pressure, tachycardia, increased LV end diastolic and systolic diameter, young age, lower BMI and NYHA functional class [17, 19].

Similar to our study finding, the majority of patients with PPCM do not achieve complete recovery of LV function as shown in other African studies. In one study of 176 patients with newly diagnosed PPCM in South Africa, complete recovery of LV function was noted in 21% of the patients at six months of follow-up [19]. A comparable proportion of patients with complete recovery of LV function was noted in our study (46.3%) and in a study conducted in Zimbabwe (45.7%) [26].

### Study limitations

This study had a short follow-up period of only six months. A longer follow-up period would have offered more information about maternal and foetal outcomes and their respective predictors. The study findings might not be representative of the general population because it was conducted in one tertiary specialised cardiology institution which is located in the capital city in the central region of Uganda. Despite these limitations, this is the first study to robustly investigate the burden of PPCM, its predictors and related maternal and foetal outcomes in Uganda and in Eastern Africa.

## Conclusion

PPCM is relatively prevalent in Uganda and is associated with multiple pregnancy gestation. In this cohort, the majority of the patients with PPCM presented with less severe disease as reflected by NYHA functional status at baseline and did not achieve full recovery of the LV function within six months of follow-up. No maternal or foetal mortality was reported.

Larger studies with longer follow-up periods are warranted to further robustly investigate long term maternal and foetal outcomes in Uganda.

## Supporting information

S1 Data Peri-partum dataset. (DTA)
